# Editorial: Biomechanical Properties of Cells and Tissues and Their Impact on Cellular Adhesion and Motility

**DOI:** 10.3389/fcell.2020.00475

**Published:** 2020-06-17

**Authors:** Claudia Tanja Mierke

**Affiliations:** Biological Physics Division, Faculty of Physics and Earth Science, Peter Debye Institute of Soft Matter Physics, University of Leipzig, Leipzig, Germany

**Keywords:** cell mechanics, deformability, stiffness, viscoelasticity, migration and invasion

Besides genetic and molecular characteristics, it has emerged that biomechanical properties of cells fulfill an important role in the process of cell adhesion and migration. Biophysical approaches and techniques to measure mechanical characteristics of cells and their surrounding tissues seems to be highly valuable for understanding of healthy and diseased conditions of cells and entire tissues. Cellular and animal model systems are helpful in enlightening the special role of cell mechanics on fundamental cellular functions such as cell adhesion and migration. A special focus on biomechanical research lies on the interaction of cell mechanical properties with nuclear mechanics and surrounding extracellular matrix mechanics. All these three types of biomechanical properties impact one another. Despite that, biomechanical properties help to shed light on the disease progression of melanoma cells as it has been reviewed in this Research Topic (Brás et al.). This special Research Topic of Frontiers in Cell and Developmental Biology includes novel research articles (Dutta et al.; Fischer et al.; Gasparski et al.; Lange et al.; Mierke et al.) and reviews (Brás et al.; Mierke; Paddillaya et al.).

The cancer cells inside a tumor experience a pronounced mechanical stimulation of the primary tumor, including a wide array of mechanical forces. While the malignant progression of tumors, such as metastasis, is highly explored, the mechanisms by which mechanical forces alter the invasive capacity of cancer cells have attracted substantially reduced awareness. Through employment of a mechanosensing invasion assay, the impact of mechanical properties on 3D motility has been explored in this Research Topic (Gasparski et al.). A special focus was set on the impact of PAK1 and its interaction with the beta-3 integrin subunit. In line with this, in another research article of this Research Topic, the role of PAK1 on the mechanical properties of mouse embryonic fibroblasts in dependence of Rac1 expression has been investigated (Mierke et al.). The alterations of the cell mechanical properties due to PAK1 and Rac1 impacted the migratory capacity of cells to invade into 3D extracellular matrices. Hence, the authors of both manuscripts proposed the hypothesis that PAK1 fulfills a prominent function in the process of biomechanical properties-based cell invasion. It would be of interest to explore whether this mechanism is (i) involved in biomechanical property-based cell invasion, (ii) is crucial in many cell and cancer types, and (iii) whether it also plays an important role in the transmigration of cancer cells through endothelial cell layers of blood or lymphatic vessels.

In a zebrafish model, gradients in cortical tension can evoke alterations in the contractile actomyosin network underneath the plasma membrane, referred to as cortical flow phenotype. The cortical flow can determine the cellular polarization and subsequently cell adhesion, including cell- cell interactions, through Par-3 signaling (Dutta et al.). An interesting question would be whether this cortical flow phenotype can also be detected in cancer cells during the formation of the primary tumor and the promotion of an invasive phenotype of specific cancer cell subtypes.

Apart from the cortical flow, cellular functions, such as cell adhesion, can be impacted by shear stress (Paddillaya et al.). It is mentioned that there is a connection between cell nucleus, cytoskeleton, plasma membrane, and extracellular matrix microenvironment. In another research article, the impact of nuclear mechanics on the invasive capacity of breast cancer cells is explored (Fischer et al.). In specific detail, the nuclear mechanics of invasive breast cancer cells was altered through the cytoskeletal mechanical properties, such as F-actin stress fibers. An intriguing and yet unresolved aspect of nuclear mechanics is whether altered nuclear mechanics can also alter the cytoskeletal mechanics.

In this Research Topic, another research article systematically investigated the interaction between actin reflux within the lamellipodium and the drift velocity of actin bundles in spatially confined and myosin activity-controlled cells (Lange et al.). In specific, also the actin bundle fusion has been investigated. A review article of this Research Topic highlighted that biomechanical properties of cells and their impact on cellular functions, such as cell adhesion and migration, may either vary among different cell types or disease types or disease states (Mierke). However, it also points out to the universal nature of specific mechanical properties of several different cell types and cancer types, which can be measured by biophysical techniques, such as the optical cell stretcher.

All articles show the complexity of different research approaches to reveal the biomechanical properties of cells and tissues. There are still some interesting facts and questions that can be addressed in future approaches, such as the impact of the surrounding microenvironment of cells, which definitely affects mechanical properties of cells. There are other crucial molecules, such as focal adhesion kinase, talin, or vinculin that affect the biomechanical properties of cells and subsequently cell adhesion and migration. Other unanswered questions include, how nuclear mechanics are altered by matrix mechanics or whether cells in turn can alter biochemically their surroundings and circumvent the impact of nuclear mechanics on cell migration and invasion. Another focus on biomechanical research will be to analyze mechanical properties of the cytoskeleton, the nucleus and the matrix microenvironment altogether in a 3D approach, where additionally also the direct impact on cell adhesion, migration and invasion can be accessed. Therefore, novel biophysical techniques, such as flow field analysis or bead-free traction force analysis in 3D, are required to measure biomechanical characteristics of migrating and resting cells in a live-cell imaging approach.

In summary, besides cytoskeletal structural arrangements, cell mechanical properties are strongly impacted by nuclear mechanics, extracellular matrix mechanics and the degree of coupling between cytoskeletal and nuclear structures as well as cytoskeletal structures and the surrounding extracellular matrix. Due to this coupling, many molecular structures can serve as mechanosensors and contribute to the cellular phenotype under healthy conditions and the malignant phenotype of cells. In future research it would be interesting to reveal whether the connection between the nuclear components and the cytoskeleton is necessary to provide the overall mechanical properties of cells or whether the mechanical phenotype relies solely on the conformational state of the nuclear chromatin and hence on the shape of the nucleus.

## Author Contributions

The author confirms being the sole contributor of this work and has approved it for publication.

## Conflict of Interest

The author declares that the research was conducted in the absence of any commercial or financial relationships that could be construed as a potential conflict of interest.

